# TIM-3 Qualifies as a Potential Immunotherapeutic Target in Specific Subsets of Patients with High-Risk Soft Tissue Sarcomas (HR-STS)

**DOI:** 10.3390/cancers15102735

**Published:** 2023-05-12

**Authors:** Luc M. Berclaz, Annelore Altendorf-Hofmann, Lars H. Lindner, Anton Burkhard-Meier, Dorit Di Gioia, Hans Roland Dürr, Alexander Klein, Markus Albertsmeier, Nina-Sophie Schmidt-Hegemann, Frederick Klauschen, Thomas Knösel

**Affiliations:** 1 Department of Internal Medicine III, University Hospital, Ludwig-Maximilians-University (LMU) Munich, 81377 Munich, Germanyanton.burkhardmeier@med.uni-muenchen.de (A.B.-M.);; 2 Department of General, Visceral and Vascular Surgery, Friedrich-Schiller University Jena, 07747 Jena, Germany; 3 Orthopaedic Oncology, Department of Orthopaedics and Trauma Surgery, University Hospital, Ludwig-Maximilians-University (LMU) Munich, 81377 Munich, Germany; 4 Department of General, Visceral and Transplantation Surgery, University Hospital, Ludwig-Maximilians-University (LMU) Munich, 81377 Munich, Germany; 5 Department of Radiation Oncology, University Hospital, Ludwig-Maximilians-University (LMU) Munich, Marchioninistr. 15, 81377 Munich, Germany; 6 Institute of Pathology, Ludwig-Maximilians-University (LMU) Munich, 81377 Munich, Germany

**Keywords:** sarcoma, tumor biomarkers, immune checkpoint inhibitors, immunotherapy, TIM-3

## Abstract

**Simple Summary:**

T cell immunoglobulin and mucin domain-containing protein 3 (TIM-3) acts as an immune checkpoint on exhausted T cells and has been associated with dismal outcomes in various solid tumors. TIM3 is currently being evaluated as an immunotherapeutic target in multiple clinical trials. Our study shows the significant tumor cell expression of TIM-3 in specific subsets of patients with high risk soft tissue sarcomas (HR-STS). We demonstrate an interaction between TIM-3, tumor infiltrating lymphocyte (TIL) counts and PD-1/PD-L1 expression in patients with HR-STS. TIM-3 could qualify as a potential immunotherapeutic target in HR-STS.

**Abstract:**

(1) Background: The expression of T cell immunoglobulin and mucin domain-containing protein 3 (TIM-3), an immune checkpoint receptor on T cells, has been associated with dismal outcomes and advanced tumor stages in various solid tumors. The blockade of TIM-3 is currently under examination in several clinical trials. This study examines TIM-3 expression in high-risk soft tissue sarcomas (HR-STS). (2) Methods: Tumor cell expression of TIM-3 on protein level was analyzed in pre-treatment biopsies of patients with HR-STS. TIM-3 expression was correlated with clinicopathological parameters including tumor-infiltrating lymphocyte (TIL) counts, programmed cell death 1 (PD-1) and programmed cell death ligand 1 (PDL-1) expression in patients with HR-STS. Survival dependent on the expression of TIM-3 was analyzed. (3) Results: TIM-3 expression was observed in 101 (56%) out of 179 pre-treatment biopsies of patients with HR-STS. TIM-3 expression was significantly more often observed in undifferentiated pleomorphic sarcomas (UPS) compared to other histological subtypes (*p* < 0.001), high TIL counts (*p* < 0.001), and high PD-1 (*p* < 0.001) and PD-L1 expression (*p* < 0.001). TIM-3 expression did not have a prognostic impact on survival in patients with HR-STS. (4) Conclusions: This is the first study to demonstrate a significant tumor cell expression of TIM-3 in specific subsets of patients with HR-STS. TIM-3 qualifies as a potential immunotherapeutic target in HR-STS.

## 1. Introduction

High-risk soft tissue sarcomas (HR-STS) are rare tumors with multiple distinct histopathological subtypes, the most common being liposarcoma, leiomyosarcoma and undifferentiated pleomorphic sarcomas (UPS). They account for approximately 1% of adult malignancies [1,2]. Despite optimal local treatment, almost half of patients will die within 5 years of their diagnosis [3,4]. In patients with advanced and metastatic soft tissue sarcomas, median survival rates range around 12–18 months [5,6,7,8]. Standard treatment for locally advanced and metastatic HR-STS is systemic chemotherapy with anthracycline-based regimens [9,10,11]. Different second- or third-line regimens, including trabectedin, or targeted therapies such as pazopanib have been approved in recent years, with only limited effects on PFS and OS [12,13]. Considering the lack of efficient therapy lines and poor survival rates, there is a great need for new systemic treatment strategies. 

While checkpoint inhibitors (CPI) revolutionized the treatment of multiple cancers with high somatic mutation rates such as melanoma and lung cancer, they have demonstrated only limited effects in sarcomas and are currently not part of international treatment guidelines [14,15,16,17,18]. T cell immunoglobulin and mucin domain-containing protein 3 (TIM-3), an emerging immune checkpoint receptor, is a member of the TIM family and was originally identified as a receptor expressed on interferon-γ-producing CD4+ and CD8+ T cells [19]. The working mechanisms of TIM-3 are not fully understood. In lymphocytes, TIM-3 is recruited to the immunological synapse on T cell activation [20]. Depending on the interplay with its interacting ligands such as CEACAM1 or lectin galectin 9, TIM-3 is differently phosphorylated and either permissive or inhibitory to T cell activation [21,22]. TIM-3 is expressed in different tumor cells, including lung cancer and melanoma [23,24]. It is co-stimulated and co-regulated with other checkpoint receptors, and the co-expression of TIM-3 with PD-1 is associated with a specific subset of particularly dysfunctional or “exhausted” T cells [22]. TIM-3+/PD-1+ cells appear to express significantly lower amounts of effector cytokines such as IFN-γ, TNF and IL-2 [25]. Both checkpoint receptors are co-regulated by immunosuppressive cytokines such as IL-27, which finally results in a diminished immune response in cancer and chronic viral infections [25,26,27]. The expression of TIM-3 was associated with a poor overall survival and advanced tumor stages in several solid malignancies, including colorectal and non-small cell lung cancer [28]. In soft tissue and bone sarcomas, TIM-3 expression in tumor-infiltrating lymphocytes (TIL) did not significantly correlate with PFS or OS in previous studies [29,30].

TIM-3 inhibition has shown promising results in pre-clinical models and is currently being evaluated as a novel immunotherapeutic approach in several clinical trials [31,32,33,34]. Clinical trials often combine TIM-3 inhibitors with checkpoint inhibitors targeting PD-1, as pre-clinical models demonstrated a synergistic effect and a better restoration of T cell responses in CPI “co-blockades” [25,35,36]. Ongoing clinical trials include NCT03446040 combining an anti-TIM-3 antibody with Nivolumab, and NCT03744468 combining anti-TIM-3 antibodies with Tislelizumab. In the present study, we analyzed the tumor cell expression of TIM-3 in a large and well-characterized cohort of HR-STS patients with long-term follow-up. We correlated our findings with clinical tumor characteristics, tumor-infiltrating lymphocyte (TIL) counts, PD-1 and PD-L1 expression status, and survival data. Our study demonstrates a significant expression of TIM-3 in specific subsets of patients with HR-STS.

## 2. Materials and Methods

### 2.1. Patient Selection

An exploratory retrospective cohort study design was chosen to address the research question. Eligible patients had pathologically confirmed high-risk soft tissue sarcoma (Tumor diameter 5 cm or larger, French Fédération Nationale des Centres de Lutte Contre le Cancer (FNCLCC) grade 2 or 3, deep to the fascia). Clinical, pathological, and outcomes data were extracted from our clinical sarcoma database. Most patients were to receive a multimodal treatment including neoadjuvant doxorubicin-based chemotherapy and regional hyperthermia (RHT), surgery, adjuvant chemotherapy + RHT and radiotherapy in select cases. Treatment continued unless disease progression or unacceptable toxic effects occurred. Follow-up was performed until December 2022.

### 2.2. Histopathology and Tissue Microarray Construction

Tumor samples originated from tumor biopsies that were taken before the initiation of neoadjuvant treatment at the Ludwig Maximilians University hospitals, Munich. In addition to the original pathology reports, microscopic findings (tumor type according to current WHO classifications and degree of differentiation) were reassessed. For tissue microarray (TMA) assembly, representative tumor areas were marked on H&E-stained slides of formalin-fixed, paraffin-embedded tumor samples from all patients according to standard procedures, and two 0.6 mm punch biopsies were taken from each sample [37]. Normal tonsillar tissue samples were used as controls on the TMA. In the end, seven tissue microarrays containing 179 pre-treatment tumor samples from 179 patients with high-grade soft tissue sarcomas (HR-STS) were constructed. 

### 2.3. TIM-3 Immunohistochemistry

For the immunohistochemical detection of TIM-3, commercially available and validated monoclonal antibodies were used (TIM-3 D5D5R, Cell Signaling Tech., Danvers, MA, USA). Antigen retrieval was carried out by heat treatment with Target Retrieval Solution Citrate (Agilent Technologies, Santa Clara, CA, USA). Staining was performed on a Ventana Benchmark XT Autostainer (Ventana Medical Systems, Tucson, AZ, USA) with a DAB+ Kit (Agilent Technologies, Santa Clara, CA, USA). All slides were counterstained with hematoxylin (Vector Laboratories, Burlingame, CA, USA). An ImmPRESS Anti-Rabbit IgG Polymer Kit was used for detection (Vector Laboratories, Burlingame, CA, USA). To exclude unspecific staining, system controls were included. Tonsillar tissue served as a positive control for immunohistochemistry. The immunostaining of cells was evaluated and scored semi-quantitatively (0 = negative; 1 = ≥5% positive and weakly stained, 2 = ≥25% positive and moderately stained, 3 = ≥50% positive and strongly stained). All immunohistochemical and pathologic evaluations were carried out independently and blinded with an experienced sarcoma pathologist (T.K.). In the case of discrepancy, the slides were reevaluated under a multiheaded microscope and a consensus was reached. 

### 2.4. TILs, PD-1 and PD-L1

Tumor-infiltrating lymphocytes (TILs), PD-1 and PD-L1 were previously investigated in our HR-STS cohort [38,39]. TILs were counted per high-power field (HPF) (400× magnification, field of view 0.237 mm^2^) in H&E-stained TMA slides. As previously described, slides were pre-treated with heat and Target Retrieval solution (S1699, Agilent, Santa Clara, CA, USA) before incubation with the monoclonal primary anti-PD-1 mouse antibody (315M; 1:80; Cell Marque, Rocklin, CA, USA) for 60 min at room temperature. The Vectastain Elite ABC HRP Kit (Vector Laboratories, Burlingame, CA, USA) and the chromogen DAB+ (Agilent) were used for detection, and Hematoxylin (Vector Laboratories) for counterstaining. For PD-L1 staining, slides were pre-treated with heat and the Epitope Retrieval Solution pH8 Novocastra (Leica Biosystems, Wetzlar, Germany) before incubation with the monoclonal primary anti-PD-L1 rabbit antibody (E1L3N; 1:50; Cell Signaling Technology, Danvers, MA, USA) for 60 min at room temperature. We used the SignalStain Boost IHC Detection Reagent (Cell Signalling Technology) and the chromogen DAB+ (Agilent) for detection according to previous studies [39].

### 2.5. Statistical Analysis

Categorical variables were tested for independence using the Chi square test. Binary variables were compared using Fisher’s Exact Test, and continuous variables were compared using *t*-tests. Logistic regression was used for univariate and multivariate analyses. The forward stepwise procedure was set to a threshold of 0.05. Data analysis was performed using SAS 9.4 (SAS Inst Inc., Cary, NC, USA). All *p*-values were based on a two-tailed hypothesis test, with values less than 0.05 considered statistically significant.

## 3. Results

### 3.1. Patient Cohort

In total, 179 patients treated between 1997 and 2019 were included in this study. The median age was 54 years (range, 18–79 years), and 87 (48.6%) patients were female. The most common histological subtypes were undifferentiated pleomorphic sarcomas (UPS) (33%), leiomyosarcomas (17%), and liposarcomas (22%). The clinicopathologic characteristics of the study cohort are summarized in Table 1.

### 3.2. TIM-3 Expression in High-Risk Soft Tissue Sarcomas (HR-STS)

**Figure 1:**
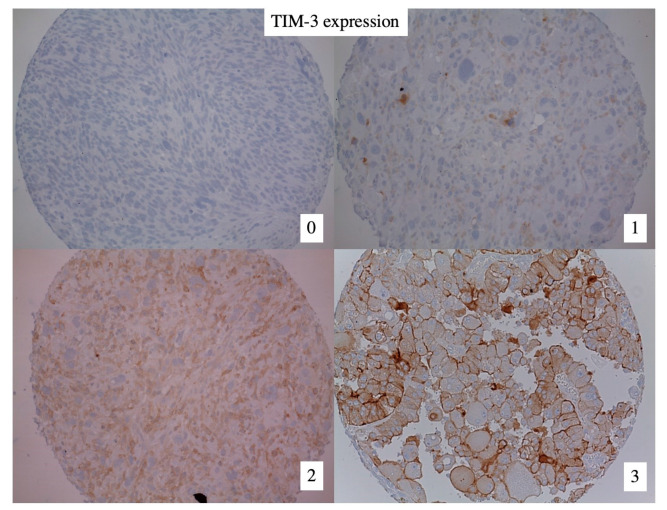
Stained tissue microarray (TMA) cores. Representative micrographs of cores on a TMA stained for TIM-3. Numbers represent semiquantitative scoring of immunostaining: 0, negative. 1–3, positive. Magnification, 20×.

TIM-3 expression was observed in 101 (56%) out of 179 pre-treatment biopsies of patients with HR-STS. Examples of immunohistochemistry staining for TIM-3 are shown in Figure 1. TIM-3 was more often positive in male than female patients (64% vs. 48%, *p* = 0.036) and associated with older age (67% vs. 47%, *p* = 0.010). TIM-3 expression was more common in undifferentiated pleomorphic sarcomas (UPS) compared to other histological subtypes (75% vs. 47%, *p* < 0.001). There was no significant association between TIM-3 expression and FNCLCC grade (*p* = 0.229). A large proportion of patients received neoadjuvant anthracycline-based chemotherapy (80%), and nearly all patients underwent R0/R1 resection (*n* = 152, 89%) (Table 2).

### 3.3. TIM-3 Expression Is Associated with TILs, PD-1 and PD-L1 Expression Status

TIM-3 expression was associated with high tumor-infiltrating lymphocyte (TIL) counts (77% vs. 43%, *p* < 0.001), high positive PD-1 (60% vs. 30%, *p* < 0.001) and positive PD-L1 expression (91% vs. 47%, *p* < 0.001). We performed a logistic regression analysis of TIM-3 expression using an inclusion approach. Sex, age, increased TIL counts, PD-L1 expression and UPS histological subtype remained statistically significant predictors of TIM-3 expression (Table 3).

### 3.4. TIM-3 Expression and Survival

**Figure 2:**
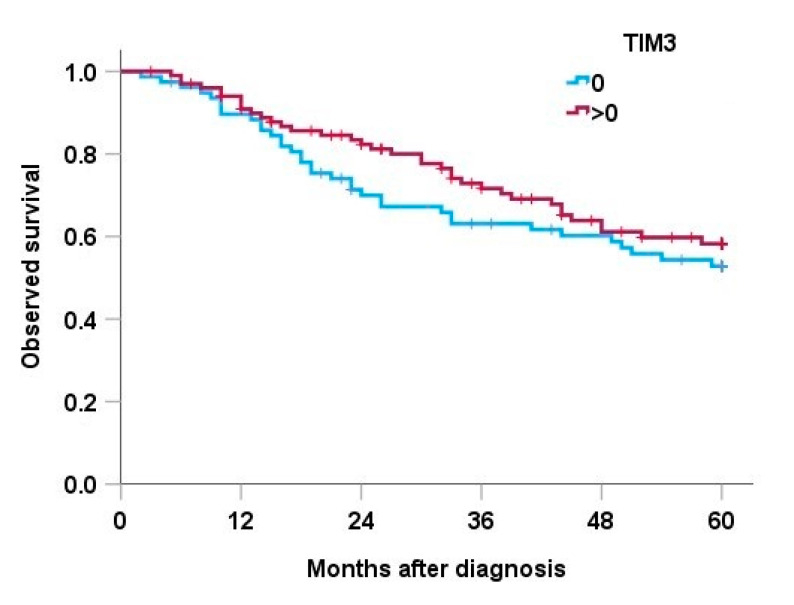
Overall survival according to TIM-3 expression.

The median follow-up duration was 119 months (95% CI 109–128 months). In total, 71 patients (40%) died within 5 years after diagnosis. Statistically significant risk factors for an unfavorable outcome in univariate survival analysis were positive surgical margins (*p* < 0.001), grade (*p* = 0.015), presence of distant metastases (*p* < 0.001) and chemotherapy (*p* = 0.010) (Table 4). Expression of TIM-3 was not associated with statistically significant changes in overall survival (*p* = 0.339) (Figure 2). 

Observed 5-year overall survival (OS) was not significantly influenced by TIM-3 expression in different histological subtypes (UPS (*p* = 0.207), leiomyosarcoma (*p* = 0.660), liposarcoma (*p* = 0.767), and other histological subtypes (*p* = 0.681)). All tested immune markers including TIM-3, PD-1, PD-L1 and tumor-infiltrating lymphocytes (TIL) did not have a statistically significant impact on 5-year OS in univariate analysis. In a multivariate Cox proportional hazards model, grade (*p* = 0.014), surgical margins (*p* < 0.001), and presence of distant metastases (*p* = 0.003) remained statistically significant independent predictors of 5-year OS. In conclusion, TIM-3 did not have a statistically significant prognostic impact on overall survival.

## 4. Discussion

To our knowledge, this is the first study to analyze the tumor cell expression of TIM-3, a novel immune checkpoint receptor and potential biomarker, in a well-characterized cohort of patients with HR-STS. TIM-3 expression was observed in 56% of patients. Our analysis indicates that patients with undifferentiated pleomorphic sarcomas (UPS), male gender, age ≥ 55 years and high expression of other immune markers (high TIL counts, positive PD-1 and PD-L1 expression) are more likely to demonstrate strong TIM-3 expression. These results remain significant in a logistic regression model, and indicate that specific subgroups of patients with HR-STS are more likely to express TIM-3.

We demonstrate the strong tumor cell expression of TIM-3 in undifferentiated pleomorphic sarcomas compared to other histological subtypes (75% vs. 47%, *p* < 0.001). UPS belong to non-translocation associated sarcomas and are associated with abundant immune infiltrates due to a higher mutational burden, higher neoantigen counts, and greater intratumoral heterogeneity compared to other entities [40]. Dancsok et al. described higher levels of PD-1, PD-L1 and TIM-3 expression on tumor-infiltrating lymphocytes in non-translocation-associated sarcomas including UPS [29]. In a study by Klaver et al., UPS had the highest fraction of PD-1+/LAG3+/TIM-3+/CD8+ T cell infiltrates, which was comparable to known “immune-dense” tumors such as malignant melanoma [41]. These findings correlate with clinical studies on immune checkpoint inhibitors in sarcomas, where UPS generally were among the best responders [14,17,42]. The strong expression of TIM-3 in UPS tumor cells supports the notion of an immunogenic signature in both tumor cells and immune infiltrates in this entity. 

Our results suggest differences in TIM-3 expression according to age and sex. Reitsema et al. have provided evidence that both age and sex modulate the expression of immune checkpoints by human T cells [43]. Interestingly, their results described a decline in PD-1 expression with age and female sex, while our results demonstrate a stronger expression of TIM-3 in male patients ≥ 55 years of age. Age-related differences in immune checkpoint expression have shown direct effects on the treatment efficacy in other tumors, including head and neck cancer or malignant melanoma [44,45]. In consequence, age- and sex-associated differences in TIM-3 expression should be considered as relevant clinical parameters in ongoing clinical trials. 

In addition to TIM-3, 60% of patients demonstrated a significant co-expression of PD-1. The expression of PD-L1 in combination with TIM-3 was observed in 91% of patients. In pre-clinical models, the co-expression of TIM-3 and PD-1 was observed in strongly dysfunctional T cells [25,27,46]. In addition, Koyama et al. demonstrated that TIM-3 can be upregulated as a result of PD-1-directed therapy [35]. With regard to these results, studies in murine models of melanoma, colorectal cancer and AML have analyzed checkpoint co-blockades, and demonstrated greater T cell responses following TIM-3 and PD-1 co-blockades compared to PD-1 inhibition alone [36,47,48]. In metastatic sarcomas, D’Angelo et al. have demonstrated increased response rates in co-blockades with anti-PD-1 and anti-CTLA4 antibodies, while anti-CTLA4 antibodies did not prove effective [17]. Our results provide an additional rationale for checkpoint co-blockades in high-risk soft tissue sarcomas, and support current clinical trials on combinations of anti-TIM-3 and anti-PD-1 antibodies in solid tumors. 

We were not able to demonstrate differences in overall survival (OS) in TIM-3+ vs. TIM-3- patients with high-risk soft tissue sarcomas (*p* = 0.339). These results are in line with previous studies on TIM-3 in soft tissue and bone sarcomas: Ligon et al. analyzed tumor-infiltrating lymphocytes in osteosarcoma pulmonary metastases and compared them with primary bone tumors. While PD-L1 and LAG3 significantly predicted progression-free survival (PFS), there was no correlation between TIM-3 status and survival [30]. In addition, Dancsok et al. were not able to correlate TIM-3 expression on tumor-infiltrating lymphocytes of soft tissue and bone sarcomas with OS or PFS [29]. In contrast, a meta-analysis conducted by Zhang et al. reported significantly shorter OS rates and advanced tumor stages in patients with positive TIM-3 expression in various solid tumors including colon cancer, gastric cancer, renal cell carcinoma and non-small cell lung cancer (NSCLC) [28]. Furthermore, Wang et al. associated TIM-3 expression with a shorter OS in esophageal squamous cell carcinoma [49]. It is currently not clear why there seems to be no significant association between survival and TIM-3 expression in high-risk soft tissue sarcomas. Possible reasons could be the large number of histological subtypes and typically small sample size in rare tumors. 

Our results demonstrate TIM-3 expression in tumor cells of patients with high-risk soft tissue sarcomas. These findings indicate that tumors with low tumor-infiltrating lymphocyte (TIL) counts can still express TIM-3 and perhaps benefit from future TIM-3 targeting therapies. Currently, there are only limited data on TIM-3 expression in tumor cells: Wiener et al. demonstrated the expression of TIM-3 in melanoma cells, and Zhuang et al. were able to detect TIM-3 in non-small cell lung cancer (NSCLC) [23,24]. In their study, TIM-3 stained positive on tumor cells in 86.7% of patients with primary NSCLC, and was associated with higher T classification and shorter OS. Interestingly, TIM-3 only stained positive in tumor cells and tumor-infiltrating lymphocytes, but not in normal (control) lung tissue, which adds to the current notion of TIM-3 playing an active role in carcinogenesis. 

In addition to the typical limitations of a retrospective study design and immunohistochemical analyses, not all patients underwent the same treatment, which could have an impact on our survival analyses. In conclusion, new systemic therapy options are needed for high-risk soft tissue sarcomas. Immunotherapeutic approaches have become a cornerstone of modern oncology, with many drugs becoming approved for a variety of tumors. This study might help us to better select the patients with HR-STS who might express higher levels of TIM-3, and therefore be candidates for potential new clinical trials.

## 5. Conclusions

To date, checkpoint inhibitors have shown only limited efficacy in patients with high-risk soft tissue sarcomas (HR-STS). Selective TIM-3 blockade has demonstrated promising results in pre-clinical trials, and acts as a potential immunotherapeutic target in combination with established checkpoint inhibitors in ongoing clinical trials. This is the first study to demonstrate a significant tumor cell expression of TIM-3 in specific subsets of patients with HR-STS. We were able to correlate TIM-3 expression with high levels of tumor-infiltrating lymphocytes and PD-1/PD-L1 expression. Our results promote the identification of potential candidates for immunotherapy in HR-STS to expand therapeutic options and move on from the current “one-size-fits-all” paradigm in the therapy of advanced HR-STS. 

## Figures and Tables

**Table 1:** Patient characteristics.

Factor	Strata	*n*	%
Total		179	100
Sex	Male	92	51
	Female	87	49
Histological subtype	UPS	59	33
	Liposarcoma	40	22
	Leiomyosarcoma	31	17
	Synovial sarcoma	18	10
	MPNST	12	7
	Angiosarcoma	5	3
	Malignant SFT	2	1
	Dediff. chondrosarcoma	3	2
	Myxofibrosarcoma	5	3
	Other	4	2
Location	Extremities	71	40
	Non-Extremities	108	60
Size of primary tumor (cm)	50–79 mm	46	26
	80–120 mm	62	35
	>120 mm	71	40
Presence of metastases	No	167	93
	Yes	12	7
FNCLCC Grade	Intermediate (G2)	89	50
	High (G3)	90	50
TIM-3 expression (Grades 0–3)	0	78	44
	1	56	31
	2	37	21
	3	8	4
Follow-up status 5 years after initial diagnosis	Alive	108	60
	Dead	71	40
Local recurrence within 5 years after R0/R1 resection	No local recurrence	91	60
	Local recurrence	61	40
Distant recurrence within 5 years after R0/R1 resection	No distant recurrence	103	68
	Distant recurrence	49	32

UPS: Undifferentiated Pleomorphic sarcoma. SFT: Solitary fibrous tumor. MPNST: Malignant peripheral nerve sheath tumor. Other: 1 rhabdomyosarcoma, 1 alveolar soft part sarcoma, 1 carcinosarcoma, 1 extraosseous osteosarcoma.

**Table 2:** Correlation between TIM3 expression and clinicopathological parameters.

Factor	Strata	Total	TIM-3 > 0		*p*-Value
		*n*	*n*	%	
All Patients		179	101	56	**--**
Sex	Male	92	59	64	**0.036**
	Female	87	42	48	
Age at initial diagnosis (years)	<55	92	43	47	**0.010**
	≥55	87	58	67	
Histological subtype	UPS	59	44	75	**<0.001**
	Liposarcoma	31	11	35	
	Leiomyosarcoma	40	26	65	
	Other	49	20	41	
Tumor Location	Extremities	71	47	66	**0.045**
	Non-extremities	108	54	50	
FNCLCC Grade	Intermediate (G2)	89	46	52	0.229
	High (G3)	90	55	61	
Surgical margins	R0	69	48	70	**0.011**
	R1	83	41	49	
	R2	14	4	29	
	No resection	13	8	62	
Chemotherapy	Yes	134	80	60	0.164
	No	45	21	47	
Radiotherapy	Yes	30	16	53	0.535
	No	106	48	45	
	Missing	43			
Regional Hyperthermia (RHT)	Yes	139	86	62	**0.007**
	No	40	15	38	
TIL counts (cells/50HPF)	0–5	108	46	43	**<0.001**
	≥6	70	54	77	
	Missing	1			
PD-1 expression	0	61	18	30	**<0.001**
	≥0	77	46	60	
	Missing	41			
PD-L1 expression	0	139	66	47	**<0.001**
	≥0	34	31	91	

**Table 3:** Multiple logistic regression model of relevant clinicopathological parameters.

Factor	Strata	Significance	Hazard Ratio	95.0% CI
Sex	Male vs. Female	0.026	2.289	(1.106–4.737)
Age	<55 vs. ≥55	0.027	1.030	(1.003–1.056)
TIL counts	0–5 vs. ≥6	0.002	3.499	(1.565–7.823)
PD-L1 expression	0 vs. >0	0.001	9.173	(2.420–34.772)
Histology	UPS vs. other subtypes	0.038	2.316	(1.046–5.128)

**Table 4:** Univariate and multivariate analysis of overall survival.

		Univariate		Multivariate	
Factor	Strata	Sig.	Hazard Ratio	Sig.	Hazard Ratio
Sex	Male vs. Female	0.366	0.806 (0.505–1.287)		
Age	1 year step	0.678	1.003 (0.987–1.020)		
Grade	G2 vs. G3	**0.015**	1.812 (1.122–2.926)	**0.014**	1.889 (1.139–3.133)
Surgical margins	R0/1 vs. R2	**<0.001**	7.310 (4.339–12.318)	**<0.001**	6.866 (3.815–12.357)
Distant metastases	M0 vs. M1	**<0.001**	4.187 (2.119–8.273)	**0.003**	3.059 (1.476–6.341)
PD-L1 expression	0 vs. >0	0.180	1.455 (0.840–2.520)	0.542	1.227 (0.636–2.364)
TIL counts	0–5 vs. ≥6	0.830	1.055 (0.649–1.713)	0.247	1.406 (0.790–2.502)
TIM3 expression	0 vs. >0	0.342	0.798 (0.501–1.271)	0.246	1.403 (0.792–2.483)
Histology	UPS vs. other	0.259	0.759 (0.470–1.226)		
Tumor location	Extremities vs. non-Extremities	0.285	1.302 (0.802–2.112)		
Chemotherapy	Yes vs. no	**0.010**	1.912 (1.168–3.129)	0.498	1.212 (0.695–2.114)
Radiotherapy	Yes vs. no	0.241	1.440 (0.783–2.647)		
Regional hyperthermia	Yes vs. no	0.749	1.091 (0.639–1.865)		
PD1 expression	0 vs > 0	0.106	1.521 (0.914–2.530)		
TIM-3 x PDL1	Both 0 vs. both >0	0.690	1.133 (0.613–2.095)		
TIL x TIM-3	TIL ≥ 6 and TIM-3 > 0 vs. TIL < 6 and TIM-3 = 0	0.599	0.848 (0.459–1.566)		
TIM-3 x PD1	Both 0 vs. both >0	0.323	1.372 (0.733–2.569)		

## Data Availability

The data presented in this study are available on specific request from the corresponding author. The data are not publicly available for reasons of data protection and data economy.

## References

[1] Brennan M.F., Antonescu C.R., Moraco N., Singer S. (2014). Lessons learned from the study of 10,000 patients with soft tissue sarcoma. Ann. Surg..

[2] Siegel R.L., Miller K.D., Jemal A. (2020). Cancer statistics, 2020. CA Cancer J. Clin..

[3] Callegaro D., Miceli R., Bonvalot S., Ferguson P., Strauss D.C., Levy A., Griffin A., Hayes A.J., Stacchiotti S., Le Pechoux C. (2016). Development and external validation of two nomograms to predict overall survival and occurrence of distant metastases in adults after surgical resection of localised soft-tissue sarcomas of the extremities: A retrospective analysis. Lancet Oncol..

[4] Kane J.M., Magliocco A., Zhang Q., Wang D., Klimowicz A., Harris J., Simko J., Delaney T., Kraybill W., Kirsch D.G. (2018). Correlation of High-Risk Soft Tissue Sarcoma Biomarker Expression Patterns with Outcome following Neoadjuvant Chemoradiation. Sarcoma.

[5] In G.K., Hu J.S., Tseng W.W. (2017). Treatment of advanced, metastatic soft tissue sarcoma: Latest evidence and clinical considerations. Ther. Adv. Med. Oncol..

[6] Nielsen O., Judson I., van Hoesel Q., le Cesne A., Keizer H., Blay J., van Oosterom A., Radford J., Svancárová L., Krzemienlecki K. (2000). Effect of high-dose ifosfamide in advanced soft tissue sarcomas. A multicentre phase II study of the EORTC Soft Tissue and Bone Sarcoma Group. Eur. J. Cancer.

[7] Maki R.G., Wathen J.K., Patel S.R., Priebat D.A., Okuno S.H., Samuels B., Fanucchi M., Harmon D.C., Schuetze S.M., Reinke D. (2007). Randomized Phase II Study of Gemcitabine and Docetaxel Compared with Gemcitabine Alone in Patients with Metastatic Soft Tissue Sarcomas: Results of Sarcoma Alliance for Research Through Collaboration Study 002. J. Clin. Oncol..

[8] Leahy M., del Muro X.G., Reichardt P., Judson I., Staddon A., Verweij J., Baffoe-Bonnie A., Jönsson L., Musayev A., Justo N. (2012). Chemotherapy treatment patterns and clinical outcomes in patients with metastatic soft tissue sarcoma. The SArcoma treatment and Burden of Illness in North America and Europe (SABINE) study. Ann. Oncol..

[9] Von Mehren M., Randall R.L., Benjamin R.S., Boles S., Bui M.M., Ganjoo K.N., George S., Gonzalez R.J., Heslin M.J., Kane J.M. (2018). Soft Tissue Sarcoma, Version 2.2018, NCCN Clinical Practice Guidelines in Oncology. J. Natl. Compr. Cancer Netw..

[10] Gronchi A., Miah A.B., Dei Tos A., Abecassis N., Bajpai J., Bauer S., Biagini R., Bielack S., Blay J.Y., Bolle S. (2021). Soft tissue and visceral sarcomas: ESMO–EURACAN–GENTURIS Clinical Practice Guidelines for diagnosis, treatment and follow-up☆. Ann. Oncol..

[11] Judson I., Verweij J., Gelderblom H., Hartmann J.T., Schöffski P., Blay J.-Y., Kerst J.M., Sufliarsky J., Whelan J., Hohenberger P. (2014). Doxorubicin alone versus intensified doxorubicin plus ifosfamide for first-line treatment of advanced or metastatic soft-tissue sarcoma: A randomised controlled phase 3 trial. Lancet Oncol..

[12] van der Graaf W.T., Blay J.-Y., Chawla S.P., Kim D.-W., Bui-Nguyen B., Casali P.G., Schöffski P., Aglietta M., Staddon A.P., Beppu Y. (2012). Pazopanib for metastatic soft-tissue sarcoma (PALETTE): A randomised, double-blind, placebo-controlled phase 3 trial. Lancet.

[13] Demetri G.D., von Mehren M., Jones R.L., Hensley M.L., Schuetze S.M., Staddon A., Milhem M., Elias A., Ganjoo K., Tawbi H. (2016). Efficacy and Safety of Trabectedin or Dacarbazine for Metastatic Liposarcoma or Leiomyosarcoma After Failure of Conventional Chemotherapy: Results of a Phase III Randomized Multicenter Clinical Trial. J. Clin. Oncol..

[14] Tawbi H.A., Burgess M., Bolejack V., Van Tine B.A., Schuetze S.M., Hu J., D’Angelo S., Attia S., Riedel R.F., Priebat D.A. (2017). Pembrolizumab in advanced soft-tissue sarcoma and bone sarcoma (SARC028): A multicentre, two-cohort, single-arm, open-label, phase 2 trial. Lancet Oncol..

[15] Ben-Ami E., Barysauskas C.M., Solomon S., Tahlil K., Malley R., Hohos M., Polson K., Loucks M., Severgnini M., Patel T. (2017). Immunotherapy with Single Agent Nivolumab for Advanced Leiomyosarcoma of the Uterus: Results of a Phase 2 Study. Cancer.

[16] Paoluzzi L., Cacavio A., Ghesani M., Karambelkar A., Rapkiewicz A., Weber J., Rosen G. (2016). Response to anti-PD1 therapy with nivolumab in metastatic sarcomas. Clin. Sarcoma Res..

[17] D’Angelo S.P., Mahoney M.R., Van Tine B.A., Atkins J., Milhem M.M., Jahagirdar B.N., Antonescu C.R., Horvath E., Tap W.D., Schwartz G.K. (2018). Nivolumab with or without ipilimumab treatment for metastatic sarcoma (Alliance A091401): Two open-label, non-comparative, randomised, phase 2 trials. Lancet Oncol..

[18] Toulmonde M., Penel N., Adam J., Chevreau C., Blay J.-Y., Le Cesne A., Bompas E., Piperno-Neumann S., Cousin S., Grellety T. (2018). Use of PD-1 Targeting, Macrophage Infiltration, and IDO Pathway Activation in Sarcomas. JAMA Oncol..

[19] Wolf Y., Anderson A.C., Kuchroo V.K. (2019). TIM3 comes of age as an inhibitory receptor. Nat. Rev. Immunol..

[20] Clayton K.L., Haaland M.S., Douglas-Vail M.B., Mujib S., Chew G.M., Ndhlovu L.C., Ostrowski M.A. (2014). T Cell Ig and Mucin Domain–Containing Protein 3 Is Recruited to the Immune Synapse, Disrupts Stable Synapse Formation, and Associates with Receptor Phosphatases. J. Immunol..

[21] van de Weyer P.S., Muehlfeit M., Klose C., Bonventre J.V., Walz G., Kuehn E.W. (2006). A highly conserved tyrosine of Tim-3 is phosphorylated upon stimulation by its ligand galectin-9. Biochem. Biophys. Res. Commun..

[22] Huang Y.-H., Zhu C., Kondo Y., Anderson A.C., Gandhi A., Russell A.F., Dougan S.K., Petersen B.-S., Melum E., Pertel T. (2015). CEACAM1 regulates TIM-3-mediated tolerance and exhaustion. Nature.

[23] Wiener Z., Kohalmi B., Pocza P., Jeager J., Tolgyesi G., Toth S., Gorbe E., Papp Z., Falus A. (2007). TIM-3 Is Expressed in Melanoma Cells and Is Upregulated in TGF-Beta Stimulated Mast Cells. J. Investig. Dermatol..

[24] Zhuang X., Zhang X., Xia X., Zhang C., Liang X., Gao L., Zhang X., Ma C. (2012). Ectopic Expression of TIM-3 in Lung Cancers. Am. J. Clin. Pathol..

[25] Sakuishi K., Apetoh L., Sullivan J.M., Blazar B.R., Kuchroo V.K., Anderson A.C. (2010). Targeting Tim-3 and PD-1 pathways to reverse T cell exhaustion and restore anti-tumor immunity. J. Exp. Med..

[26] Zhu C., Sakuishi K., Xiao S., Sun Z., Zaghouani S., Gu G., Wang C., Tan D.J., Wu C., Rangachari M. (2015). An IL-27/NFIL3 signalling axis drives Tim-3 and IL-10 expression and T-cell dysfunction. Nat. Commun..

[27] Fourcade J., Sun Z., Pagliano O., Chauvin J.-M., Sander C., Janjic B., Tarhini A.A., Tawbi H.A., Kirkwood J.M., Moschos S. (2014). PD-1 and Tim-3 Regulate the Expansion of Tumor Antigen–Specific CD8+ T Cells Induced by Melanoma Vaccines. Cancer Res..

[28] Zhang Y., Cai P., Liang T., Wang L., Hu L. (2017). TIM-3 is a potential prognostic marker for patients with solid tumors: A systematic review and meta-analysis. Oncotarget.

[29] Dancsok A.R., Setsu N., Gao D., Blay J.-Y., Thomas D., Maki R.G., Nielsen T.O., Demicco E.G. (2019). Expression of lymphocyte immunoregulatory biomarkers in bone and soft-tissue sarcomas. Mod. Pathol..

[30] Ligon J.A., Choi W., Cojocaru G., Fu W., Hsiue E.H.-C., Oke T.F., Siegel N., Fong M.H., Ladle B., Pratilas C.A. (2021). Pathways of immune exclusion in metastatic osteosarcoma are associated with inferior patient outcomes. J. Immunother. Cancer.

[31] Harding J.J., Patnaik A., Moreno V., Stein M., Jankowska A.M., de Mendizabal N.V., Liu Z.T., Koneru M., Calvo E. (2019). A phase Ia/Ib study of an anti-TIM-3 antibody (LY3321367) monotherapy or in combination with an anti-PD-L1 antibody (LY3300054): Interim safety, efficacy, and pharmacokinetic findings in advanced cancers. J. Clin. Oncol..

[32] Ahn M. (2018). MS28.02 Combination IO + IO. J. Thorac. Oncol..

[33] Murtaza A., Laken H., Correia J.D.S., McNeeley P., Altobell L., Zhang J., Vancutsem P., Wilcoxen K., Jenkins D. (2016). Discovery of TSR-022, a novel, potent anti-human TIM-3 therapeutic antibody. Eur. J. Cancer.

[34] Burugu S., Dancsok A.R., Nielsen T.O. (2017). Emerging targets in cancer immunotherapy. Semin. Cancer Biol..

[35] Koyama S., Akbay E.A., Li Y.Y., Herter-Sprie G.S., Buczkowski K.A., Richards W.G., Gandhi L., Redig A.J., Rodig S.J., Asahina H. (2016). Adaptive resistance to therapeutic PD-1 blockade is associated with upregulation of alternative immune checkpoints. Nat. Commun..

[36] Ngiow S.F., von Scheidt B., Akiba H., Yagita H., Teng M.W.L., Smyth M.J. (2011). Anti-TIM3 Antibody Promotes T Cell IFN-γ–Mediated Antitumor Immunity and Suppresses Established Tumors. Cancer Res..

[37] Knösel T., Emde A., Schlüns K., Chen Y., Jürchott K., Krause M., Dietel M., Petersen I. (2005). Immunoprofiles of 11 Biomarkers Using Tissue Microarrays Identify Prognostic Subgroups in Colorectal Cancer. Neoplasia.

[38] Orth M.F., Buecklein V.L., Kampmann E., Subklewe M., Noessner E., Cidre-Aranaz F., Romero-Pérez L., Wehweck F.S., Lindner L., Issels R. (2020). A comparative view on the expression patterns of PD-L1 and PD-1 in soft tissue sarcomas. Cancer Immunol. Immunother..

[39] Albertsmeier M., Altendorf-Hofmann A., Lindner L.H., Issels R.D., Kampmann E., Dürr H.-R., Angele M.K., Klauschen F., Werner J., Jungbluth A.A. (2022). VISTA in Soft Tissue Sarcomas: A Perspective for Immunotherapy?. Cancers.

[40] Thorsson V., Gibbs D.L., Brown S.D., Wolf D., Bortone D.S., Ou Yang T.-H., Porta-Pardo E., Gao G.F., Plaisier C.L., Eddy J.A. (2018). The Immune Landscape of Cancer. Immunity.

[41] Klaver Y., Rijnders M., Oostvogels A., Wijers R., Smid M., Grünhagen D., Verhoef K., Sleijfer S., Lamers C., Debets R. (2020). Differential quantities of immune checkpoint-expressing CD8 T cells in soft tissue sarcoma subtypes. J. Immunother. Cancer.

[42] Toulmonde M., Lucchesi C., Verbeke S., Crombe A., Adam J., Geneste D., Chaire V., Laroche-Clary A., Perret R., Bertucci F. (2020). High throughput profiling of undifferentiated pleomorphic sarcomas identifies two main subgroups with distinct immune profile, clinical outcome and sensitivity to targeted therapies. Ebiomedicine.

[43] Reitsema R.D., Cadena R.H., Nijhof S.H., Abdulahad W.H., Huitema M.G., Paap D., Brouwer E., Boots A.M.H., Heeringa P. (2020). Effect of age and sex on immune checkpoint expression and kinetics in human T cells. Immun. Ageing.

[44] Kugel C.H., Douglass S.M., Webster M.R., Kaur A., Liu Q., Yin X., Weiss S.A., Darvishian F., Al-Rohil R.N., Ndoye A. (2018). Age Correlates with Response to Anti-PD1, Reflecting Age-Related Differences in Intratumoral Effector and Regulatory T-Cell Populations. Clin. Cancer Res..

[45] Daste A., Domblides C., Gross-Goupil M., Chakiba C., Quivy A., Cochin V., de Mones E., Larmonier N., Soubeyran P., Ravaud A. (2017). Immune checkpoint inhibitors and elderly people: A review. Eur. J. Cancer.

[46] Jin H.-T., Anderson A.C., Tan W.G., West E.E., Ha S.-J., Araki K., Freeman G.J., Kuchroo V.K., Ahmed R. (2010). Cooperation of Tim-3 and PD-1 in CD8 T-cell exhaustion during chronic viral infection. Proc. Natl. Acad. Sci. USA.

[47] Liu J., Zhang S., Hu Y., Yang Z., Li J., Liu X., Deng L., Wang Y., Zhang X., Jiang T. (2016). Targeting PD-1 and Tim-3 Pathways to Reverse CD8 T-Cell Exhaustion and Enhance Ex Vivo T-Cell Responses to Autologous Dendritic/Tumor Vaccines. J. Immunother..

[48] Zhou Q., Munger M.E., Veenstra R.G., Weigel B.J., Hirashima M., Munn D.H., Murphy W.J., Azuma M., Anderson A.C., Kuchroo V.K. (2011). Coexpression of Tim-3 and PD-1 identifies a CD8+ T-cell exhaustion phenotype in mice with disseminated acute myelogenous leukemia. Blood.

[49] Wang P., Chen Y., Long Q., Li Q., Tian J., Liu T., Wu Y., Ding Z. (2021). Increased coexpression of PD-L1 and TIM3/TIGIT is associated with poor overall survival of patients with esophageal squamous cell carcinoma. J. Immunother. Cancer.

